# Influence of cerebrospinal fluid drainage in the first days after aneurysm rupture on the severity of early brain injury following aneurysmal subarachnoid hemorrhage

**DOI:** 10.1007/s00701-024-06131-w

**Published:** 2024-05-28

**Authors:** Sheri Tuzi, Beate Kranawetter, Onnen Moerer, Veit Rohde, Dorothee Mielke, Vesna Malinova

**Affiliations:** 1 Department of Neurosurgery, University Medical Center Göttingen, Robert-Koch-Straße 40, 37075 Göttingen, Germany; 2 Department of Anesthesiology, University Medical Center Göttingen, Göttingen, Germany

**Keywords:** Early brain injury, Cerebrospinal fluid drainage, Subarachnoid hemorrhage

## Abstract

**Purpose:**

Progressive cerebral edema with refractory intracranial hypertension (ICP) requiring decompressive hemicraniectomy (DHC) is a severe manifestation of early brain injury (EBI) after aneurysmal subarachnoid hemorrhage (aSAH). The purpose of the study was to investigate whether a more pronounced cerebrospinal fluid (CSF) drainage has an influence on cerebral perfusion pressure (CPP) and the extent of EBI after aSAH.

**Methods:**

Patients with aSAH and indication for ICP-monitoring admitted to our center between 2012 and 2020 were retrospectively included. EBI was categorized based on intracranial blood burden, persistent loss of consciousness, and SEBES (Subarachnoid Hemorrhage Early Brain Edema Score) score on the third day after ictus. The draining CSF and vital signs such as ICP and CPP were documented daily.

**Results:**

90 out of 324 eligible aSAH patients (28%) were included. The mean age was 54.2 ± 11.9 years. DHC was performed in 24% (22/90) of patients. Mean CSF drainage within 72 h after ictus was 168.5 ± 78.5 ml. A higher CSF drainage within 72 h after ictus correlated with a less severe EBI and a less frequent need for DHC (*r*=-0.33, *p* = 0.001) and with a higher mean CPP on day 3 after ictus (*r* = 0.2351, *p* = 0.02).

**Conclusion:**

A more pronounced CSF drainage in the first 3 days of aSAH was associated with higher CPP and a less severe course of EBI and required less frequently a DHC. These results support the hypothesis that an early and pronounced CSF drainage may facilitate blood clearance and positively influence the course of EBI.

## Introduction

Aneurysmal subarachnoid hemorrhage (aSAH) is a severe cerebrovascular disease with a mortality rate of 35% [14]. After an aneurysm ruptures, blood flows into the subarachnoid space and blocks the cerebrospinal fluid (CSF) flow, leading to an acute increase in intracranial pressure (ICP), which is mainly responsible for the high early mortality of aSAH. A plethora of pathological processes triggered by aneurysm rupture occur within the first 72 h (referred to as early brain injury [EBI]) including increased ICP, impaired cerebral autoregulation, oxidative stress, inflammation, and disruption of the blood-brain barrier [1, 14, 15, 17]. These events can lead to severe brain edema, decreased cerebral perfusion, and neuronal injury, setting the stage for subsequent complications. Blood degradation induces a release of pro-inflammatory cytokines and free radicals into the CSF further aggravating brain edema and neuronal damage [26]. Enhancing natural clearance mechanisms could reduce brain damage and improve the prognosis of aSAH. The CSF turnover represents one crucial blood clearance mechanism and plays an important role in removing the hematoma and blood degradation products from the subarachnoid space after aSAH [22]. Previous studies have reported an increase of CSF production and flow rates early on after aSAH, contributing to the initial blood clearance [5, 27]. While a correlation between the blood amount within the subarachnoid/intraventricular spare with the incidence of delayed cerebral ischemia (DCI) has been previously demonstrated [19, 25], the role of early CSF drainage on blood clearance and subsequently on the extent of EBI and following complications has not been defined yet. CSF drainage offers benefits not only in reducing ICP, but also in improving cerebral perfusion and minimizing secondary complications [3]. The objectives of this study were: 1-to evaluate the impact of early CSF drainage within the first 72 h after ictus on blood clearance; 2-to investigate whether a more pronounced CSF drainage reduces the risk of developing progressive brain edema requiring decompressive hemicraniectomy (DHC) and supports the maintenance of sufficient cerebral perfusion after aSAH. The hypothesis was that a more pronounced CSF drainage within the first 72 h after aneurysm rupture results into a faster blood clearance and, therefore, reduces the need for DHC and leads to a higher cerebral perfusion pressure (CPP) after aSAH.

## Methods

In this retrospective observational study, we screened all consecutive patients with aSAH treated at our center from 2012 to 2020. A cranial computer tomography (CCT), computed tomography angiography (CTA), and/or digital subtraction angiography (DSA) confirmed the presence of aSAH. Only adult patients requiring ICP monitoring with the possibility to calculate CPP were included in the study. An ICP monitoring via an intraparenchymal probe was established in all high grade aSAH patients who were comatose/sedated. A Codman® ICP monitor was used.

Data concerning the baseline characteristics of the study population were obtained by reviewing the patient records and/or were extracted from the continuous documentation system at the intensive care unit (ICU). The CSF was drained either using an external ventricular drainage (EVD) or lumbar drainage (LD). The decisions concerning the indication for placing a CSF drainage and the choice of CSF drainage (LD vs. EVD) are based on a management protocol for aSAH patients, which was previously defined and established at our center. The decision to insert a CSF drainage was made based on clinical (headache) and radiological criteria (signs of CSF circulation disturbance). At our center, a LD was primarily placed in patients with lower grade aSAH (WFNS I-III), where patients with a higher aSAH grade (WFNS IV-V) primarily received an EVD. The CSF drainage was continuously open in all patients, independently of the presence of brain edema or intracerebral hematoma. The CSF drainage volume was not intentionally reduced in patients with brain edema. After the insertion of the CSF drainage, the drainage was kept open at the level of external acoustic meatus i.e., 0 mm Hg. The daily CSF drainage was documented in every patient, who had a CSF drain (EVD or LD). The presence of brain edema was semi-quantitatively assessed using the Subarachnoid Hemorrhage Early Brain Edema Score (SEBES) [1]. This score measures cerebral edema on CCT using a scale of 0 to 4 points based on the presence or absence of visible sulci. The amount of blood within the subarachnoid and intraventricular space was assessed using the Hijdra score [9] and the Le Roux score [11], respectively. The Hijdra score evaluates the amount of blood in each of the ten basal cisterns and fissures, with a maximum score of 30 points depending on the amount of blood present in each cistern: 0 points = no blood, 1 point = small amount of blood, 2 points = moderate amount of blood, and 3 points = full cistern of blood. The Le Roux score assesses the amount of blood in each of the four ventricles. It has a total possible score of 16 points: 0 points = no blood, 1 point = a trace of blood, 2 points = less than half of the ventricles filled with blood, 3 points = more than half of the ventricles filled with blood, and 4 points = complete filling of the ventricles with blood, resulting in expansion. The radiological assessment was done by a medical student (S.T.) and a neurosurgical resident (B.K.), who were blinded for CSF drainage. In case the grading has provided an incongruent result, the grading was revised by a consultant neurosurgeon (V.M.). Additionally, the reliability of the assessments was evaluated by a consultant neurosurgeon (V.M.) on a sample basis.

### Primary and secondary endpoints

The need for DHC due to progressive global cerebral edema (GCE) and refractory ICP was regarded as the most severe manifestation of EBI within the first 72 h after aSAH, that was the primary endpoint of the study. At our center, a stepwise concept is pursued during the decision-making process concerning the indication for DHC based on an established management protocol for aSAH patients. The decision for performing a DHC was made in patients with a refractory increase in ICP despite of conservative measures for ICP reduction such as neuroprotective sedation, mild hyperventilation, administration of hyperosmolar agents i.e., Mannitol, and despite of a CSF drainage.

Secondary endpoint was the early blood clearance dependent on the CSF drainage. The intracranial blood burden was semi-quantitatively assessed at two time points, on admission as well as 72 h after aneurysm rupture. The early blood clearance was evaluated as the difference between the initial intracranial blood burden and the blood burden 72 h after ictus. ICP was monitored using a probe placed within the right frontal lobe and mean arterial pressure (MAP) was continuously measured via arterial line. Cerebral perfusion pressure (CPP) was continuously calculated according to the equation CPP = MAP-ICP. Other secondary endpoints were the development of cerebral vasospasm and DCI. Cerebral vasospasm was detected by measuring the blood flow velocity of the middle cerebral artery using a transcranial Doppler ultrasound (TCD) with a cutoff value of > 120 cm/s. Delayed infarctions were defined as newly diagnosed cerebral infarctions after excluding treatment-associated infarctions by performing a CT scan within 24 h after aneurysm occlusion.

### Statistical analysis

The data was analyzed using the GraphPad Prism software (Version 10, GraphPad Software, San Diego, CA, USA) employing both descriptive and inferential statistics. Frequency and percentages were used to represent discrete, ordinal, and binary variables, while continuous variables were expressed as mean ± standard deviation. Correlation and simple logistic regression tests were run for each endpoint with dichotomous variables. For continuous variables, correlation and simple linear regression tests were applied. To display the relationship between sensitivity and specificity and estimate the tests’ overall diagnostic accuracy, receiver operatic characteristic curve (ROC curve) and area under the curve (AUC) analyses were conducted. Simple logistic regression was used to determine the relation between higher CSF drainage and DHC. Simple logistic regression was used to determine the relation between higher CSF drainage with TCD-vasospasm and DCI.

## Results

### Baseline characteristics of the study population

The inclusion criteria were met by 90 out of 324 consecutive aSAH patients. Mean age of the study population was 54.2 ± 11.9 years, 64% (54/90) of whom were female. In 83% (74/90) of the patients the aneurysm was located within the anterior circulation. The aneurysm occlusion was performed by clipping in 56% (50/90) and by coiling in 44% (40/90) of included patients. The baseline characteristics are summarized in Table 1.

**Table 1:** Baseline characteristics

Parameters	Values
Number of patients	90
Mean age ± SD (range) in years	54.2 ± 11.9 (28–80)
Sex - Male (%) - Female (%)	32/90 (36%) 58/90 (64%)
WFNS grade - WFNS I-III - WFNS IV-V	32/90 (36%) 58/90 (64%)
Fisher grade - Grade 3–4	90/90 (100%)
Aneurysm location - Anterior circulation - Posterior circulation	74/90 (83%) 16/90 (17%)
Aneurysm occlusion - Clipping - Coiling	50/90 (56%) 40/90 (44%)
CSF drainage - EVD - LD - EVD + LD	31/90 (34%) 7/90 (8%) 52/90 (58%)
Mean duration of CSF drainage in days - EVD - LD - EVD + LD	11.7 ± 7.1 6.7 ± 7.4 17.9 ± 9.0
TCD-vasospasm - Yes - No	67/90 (74%) 23/90 (26%)
Delayed cerebral infarction - Yes - No	25/90 (28%) 65/90 (72%)
Functional outcome (mRS) - mRS ≤ 2 - mRS > 2	50/90 (56%) 40/90 (44%)

### Blood clearance dependent on the CSF drainage

**Fig. 1:**
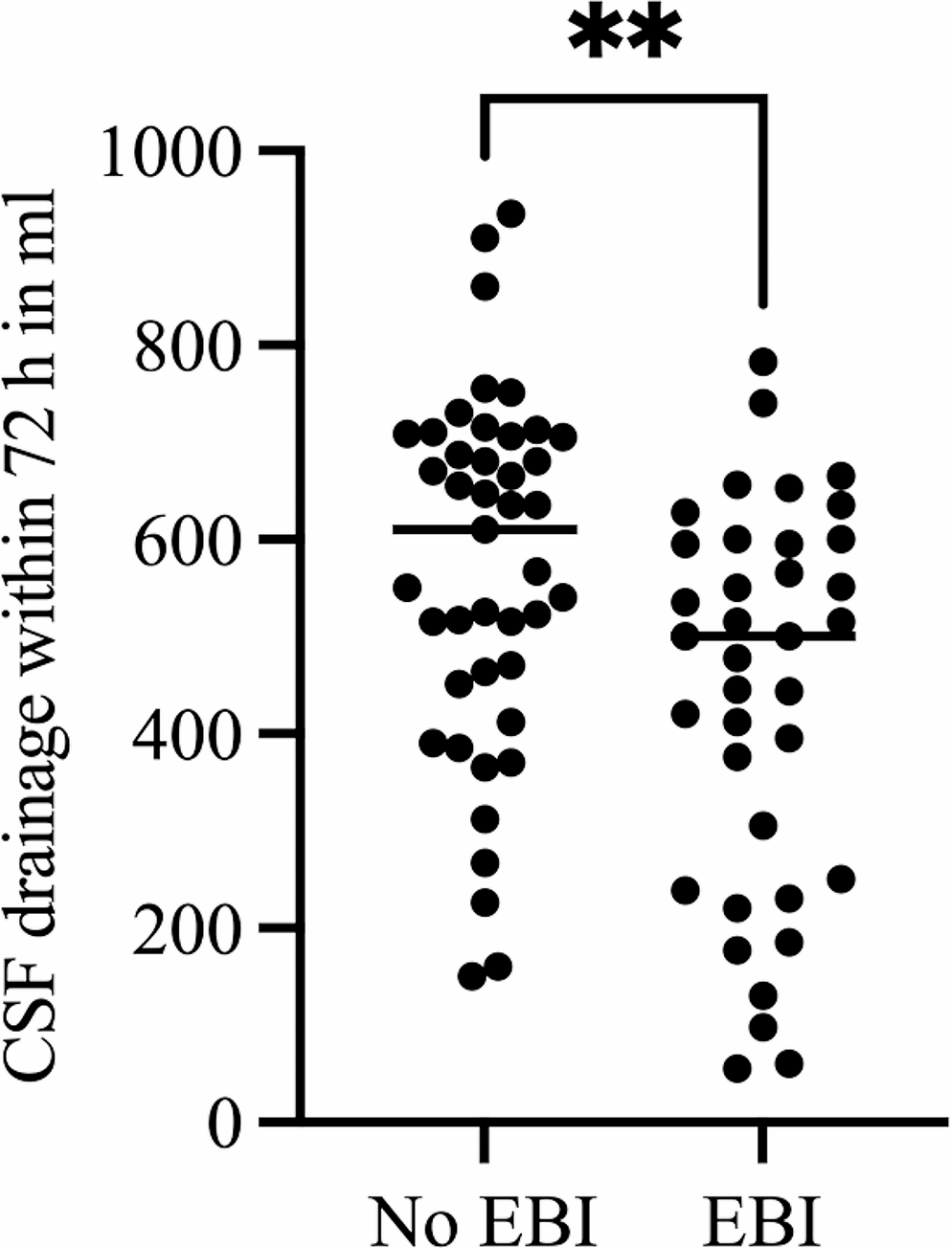
Cerebrospinal fluid (CSF) drainage within the first 72 h after aneurysm rupture with significantly lower CSF diversion rates in patients with early brain injury (EBI) compared to those without EBI (*p* = 0.003)

The mean score for overall intracranial blood burden on admission was 24 ± 9 points, that was significantly reduced three days after ictus with mean overall intracranial blood burden score of 13 ± 8 points 72 h after ictus (*p* < 0.0001), Fig. 1. The mean relative blood clearance within 72 h was 49.8% ± 26.3%. Mean early CSF drainage within the first three days was 169 ± 78 ml. Linear regression analysis revealed a significant association of higher mean CSF drainage within the first three days with a higher difference of the overall blood burden score (*p* = 0.03) and the Hijdra score (*p* = 0.01), while the Le Roux showed no significant association with early CSF drainage. On the contrary, the CSF drainage volume was not associated with the WFNS grade, the Fisher grade, and the initial Hijdra score in the linear regression analysis.

### Impact of early CSF drainage on progressive brain edema and ischemic complications

**Fig. 2:**
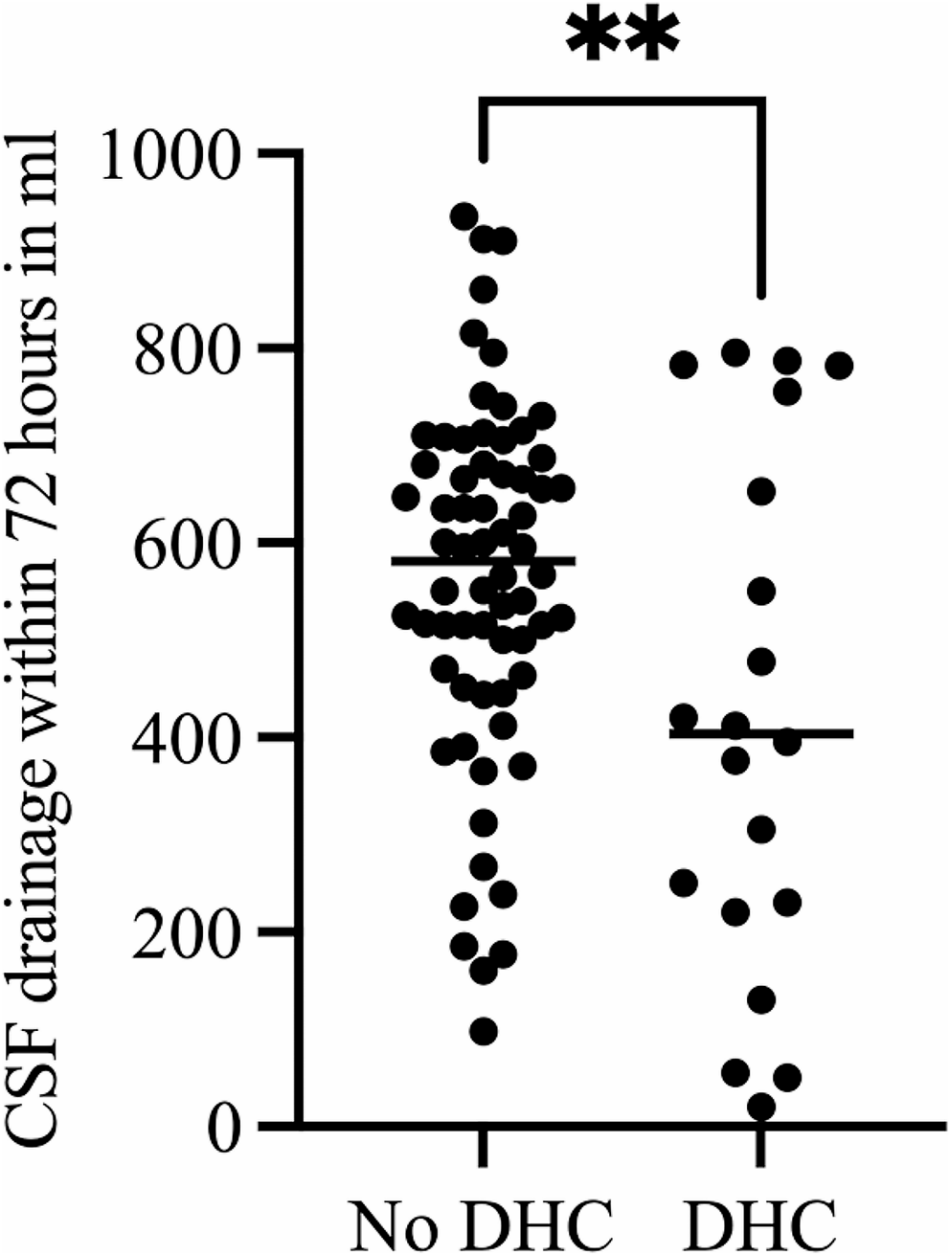
Cerebrospinal fluid (CSF) drainage within the first 72 h after aneurysm rupture with significantly lower CSF diversion rates in patients with progressive brain edema requiring decompressive hemicraniectomy (DHC) compared to those without DHC (*p* = 0.001)

**Fig. 3:**
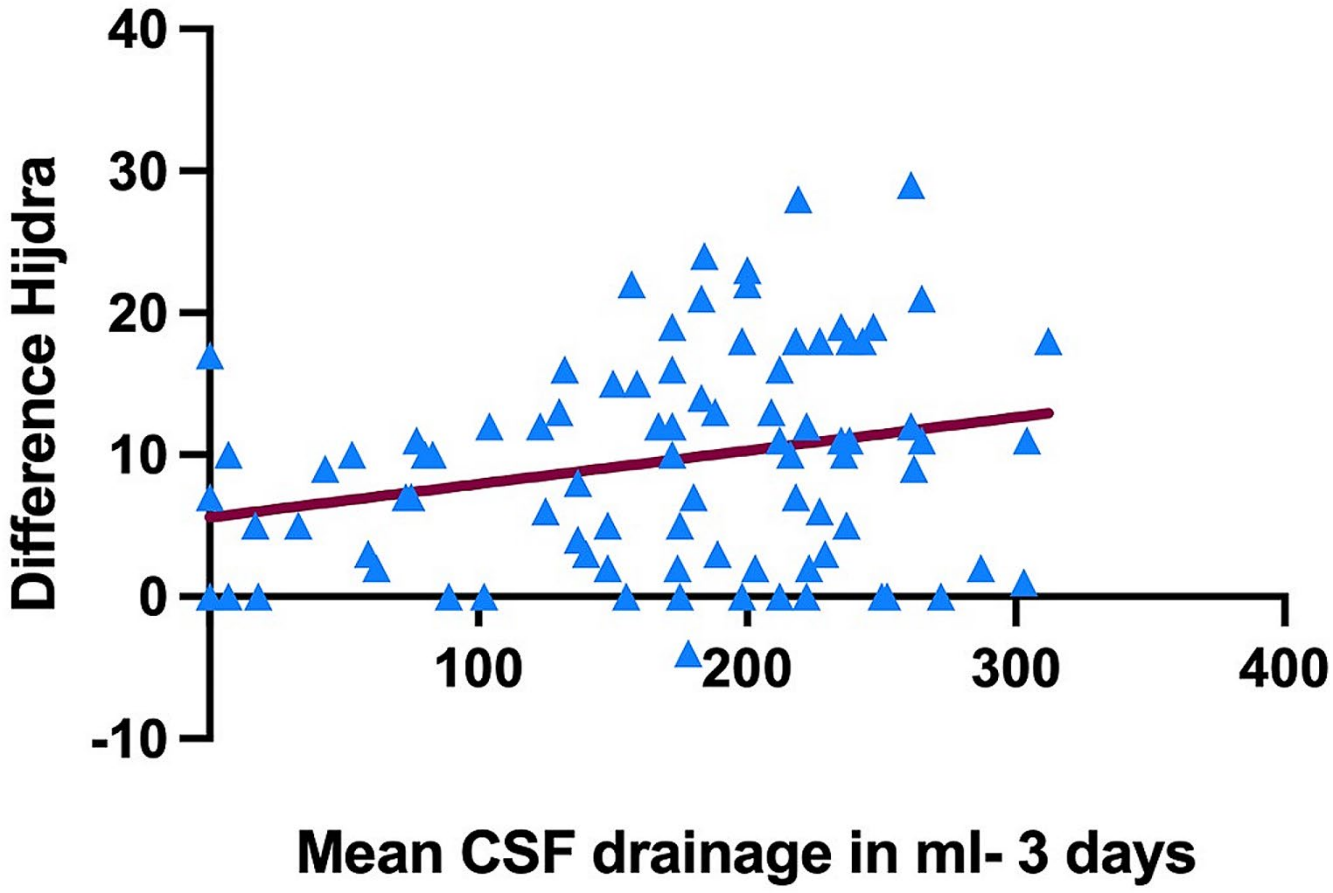
Higher cerebrospinal fluid (CSF) drainage within the first 72 h after aneurysm rupture leading to higher blood clearance within the subarachnoid space as assessed by applying the Hijdra score on admission and after 72 h (difference Hijdra score) (*p* = 0.02)

The CSF drainage volume was not associated with the presence of brain edema on admission (Regression coefficient 2.58, 95%CI 1.82 to 3.33, *p* = 0.252). The regression analysis revealed a trend to higher CSF drainage volumes in patients with larger midline shift on admission, but the difference did not reach statistical significance ((Regression coefficient 3.48, 95%CI 1.36 to 5.60, *p* = 0.063). Progressive brain edema requiring DHC developed in 24% (22/90) of patients studied. A higher mean early CSF drainage negatively correlated with progressive brain edema requiring DHC (*r*=-0.32, *p* = 0.001), Fig. 2. Mean daily CSF drainage of more than 156 ml was associated with five-fold lower risk for developing progressive brain edema requiring DHC (OR = 5.2, *p* = 0.002) (Fig. 3). Patients with a mean early CSF drainage of > 156 ml had a significantly higher average CPP within the first three days after ictus compared to those with a CSF drainage of ≤ 156 ml (*p* = 0.005). TCD-vasospasm was detected in 50% (45/90) of the patients, and 28% (25/90) developed DCI. A higher CSF drainage correlated with a lower incidence of TCD-vasospasm (*r*=-29, *p* = 0.02), and of DCI (*r*=-26, *p* = 0.02). The type of CSF drainage did not correlate with the need for DHC or with DCI.

## Discussion

In this observational study, a higher CSF drainage with enhanced blood clearance within the first three days after aSAH was associated with less frequent need for DHC due to progressive edema as well as with lower incidence of TCD-vasospasm and DCI. A faster reduction of intracranial blood burden ameliorating toxic effects of blood degradation products, hence, preventing severe EBI could be the explanation for these findings. In reverse conclusion, enhanced blood clearance could represent a promising therapeutic target to reduce the extent of EBI and avoid secondary complications.

### Mechanisms of reducing early brain injury by enhancing early blood clearance

The amount of blood in the subarachnoid space affects the severity of EBI and overall morbidity and mortality after aSAH [16]. An increase in ICP after aneurysm rupture leads to a decrease in CPP with values below 50 mmHg leading to global ischemia [21]. Perfusion disturbances after aSAH carry the risk of developing cerebral infarction with permanent neurological deficits. To ensure adequate cerebral perfusion, CPP values of over 70 mmHg are recommended in current guidelines [10]. Blood degradation products lead to oxidative stress with release of free radicals that could harm endothelial and smooth muscle cells while reducing nitric oxide (NO) production and release [2, 16]. Further pathophysiological mechanisms like alteration of vasoreactivity with consecutive vasoconstriction and activation of the inflammation cascade are additionally triggered by blood degradation [4, 13]. Hemoglobin plays an important role, as high concentrations of extracellular hemoglobin exhibit pro-inflammatory effects [2, 16]. Furthermore, the induction of microglial hemoxygenase-1 has been found to mediate blood clearance and to attenuate brain injury and vasospasm in experimental models [6]. Additionally, a correlation of the microglial hem oxygenase expression with the intracranial blood volume was reported [6, 20]. Global cerebral edema (GCE) can result from various pathophysiological processes, including blood degradation and neuroinflammation [8]. Progressive brain edema represents a severe complication of EBI with high risk for lethal outcome and often requires surgical intervention like DHC. A positive correlation between the amount of subarachnoid blood and the occurrence of cerebral vasospasm was previously reported as one of the main contributors of DCI [23]. A recent study by Reilly et al., 2018, showed that the initial subarachnoid clot volume and clot clearance per day can predict vasospasm [12, 18]. These findings underline the idea that CSF drainage and higher blood clearance may be helpful in reducing the incidence of vasospasm and DCI. The results of our study support the hypothesis, that higher early CSF drainage is associated with less severe EBI and lower risk for secondary complications in a clinical setting by enhancing blood clearance. Early CSF drainage via LD has been shown to lead to better functional outcome after aSAH in a randomized controlled trial (EARLYDRAIN) [24]. Although, one may assume a possible relation of these findings with enhanced blood clearance, the causality question remains unanswered. The findings of our study with higher CPP values and lower incidence of progressive brain edema needing DHC and DCI in patients with higher CSF drainage rates and enhanced blood clearance within the first three days after ictus are supporting the above-mentioned hypothesis. In a post-hoc analysis of the EARLYDRAIN trial, higher ICP values were found in patients with unfavorable outcome. However, the ICP values did not show a correlation with the CSF drainage volumes via EVD in that study [7]. On the contrary, the total CSF drainage volume and the CSF drainage volume via LD was associated with favorable outcome after adjusting for aSAH severity [7]. Higher CSF drainage volumes were associated with lower ICP values and with less frequent need for DHC due to refractory ICP increase in our study, which was not dependent on the type of CSF drainage. While our study had a focus on the impact of the CSF drainage in the first days after aneurysm rupture, this was not the case in the post-hoc analysis of the EARLYDRAIN trial, which may be the explanation from the partially differing findings. A CSF drainage exceeding 156 ml per day in the first three days resulted in higher CPP values was associated with lower incidence of secondary complications. A higher CSF drainage led to higher blood clearance with reduction of overall blood burden within the first three days after ictus. More enhanced drainage significantly affected the amount of blood in the basal cisterns and its difference upon admission and day three, according to the Hijdra score. These findings are in line with the results of a recently published study [25].

### Limitations

This study, while providing valuable insights, has a few limitations that must be taken into consideration. Firstly, this is a retrospective study, so there are inherent limitations due to the study design. Due to the retrospective study design, the study cannot confirm a causality of the effects. Secondly, the sample size in this study is relatively small, which could increase the risk of bias and limit the generalizability of the results. However, the study covers a consecutive cohort of patients who received standardized treatment at our center according to a predefined treatment protocol. Future studies using more advanced techniques with automated measurement of blood volume could help to overcome this limitation. Despite these limitations, this study provides important insights into the potential effectiveness of the treatment and should be considered in future research on the topic.

## Conclusions

A more pronounced CSF drainage within 72 h after aneurysm rupture was associated with higher CPP values and a less severe course of EBI that required DHC less frequently. The findings support the hypothesis that an early and pronounced CSF drainage may not only facilitate blood clearance but positively influence the course of EBI. These findings need further evaluation in a prospective study.

## Data Availability

All generated and analyzed data are presented in the manuscript.
